# Hypoimmune platforms: from rejection to immune evasion and regulatory implications

**DOI:** 10.3389/ti.2026.16845

**Published:** 2026-08-25

**Authors:** Francesco Campo, Maria Irene Bellini, Hanne Scholz, Lorenzo Piemonti, Antonio Citro

**Affiliations:** 1 Diabetes Research Institute, IRCCS San Raffaele Hospital, Milan, Italy; 2 Department of Surgery, Sapienza Università di Roma, Rome, Italy; 3 Department of Transplant Medicine, Oslo University Hospital, Oslo, Norway; 4 Vita‐Salute San Raffaele University, Milan, Italy

**Keywords:** genome editing, hypoimmune, islet transplantation, regulatory, xenotransplantation

## Abstract

The growing gap between organ demand and clinical availability has renewed interest in immune-evasive graft strategies, yet rejection and lifelong immunosuppression remain major barriers to durable success. Advances in genome editing enable immune-evasive cell platforms designed to avoid immune recognition while replacing missing function in allogeneic settings. This review summarizes current strategies for engineering immune-evasive grafts that simultaneously suppress adaptive and innate immune responses. We discuss how coordinated modulation of antigen presentation and immune checkpoint pathways can protect transplanted allogeneic cells and tissues from T, NK, and macrophage-mediated rejection. We also present the emerging concept of integrating hypoimmune engineering with genetically modified porcine donors, where extensive genome editing has reduced, but not eliminated, xenogeneic immune barriers. Combining donor genome modification with immune-evasive graft design represents a promising conceptual advance toward xenograft survival, though whether full elimination of systemic immunosuppression is achievable remains to be established clinically. We further examine how the regulatory landscape for these products is evolving across major jurisdictions, and how differences in approval pathways, manufacturing standards, and long-term surveillance requirements shape the path to clinical translation. Finally, we outline the safety considerations and remaining limitations in immune evasion that must be addressed to enable clinical implementation.

## Graphical Abstract

**Figure:**
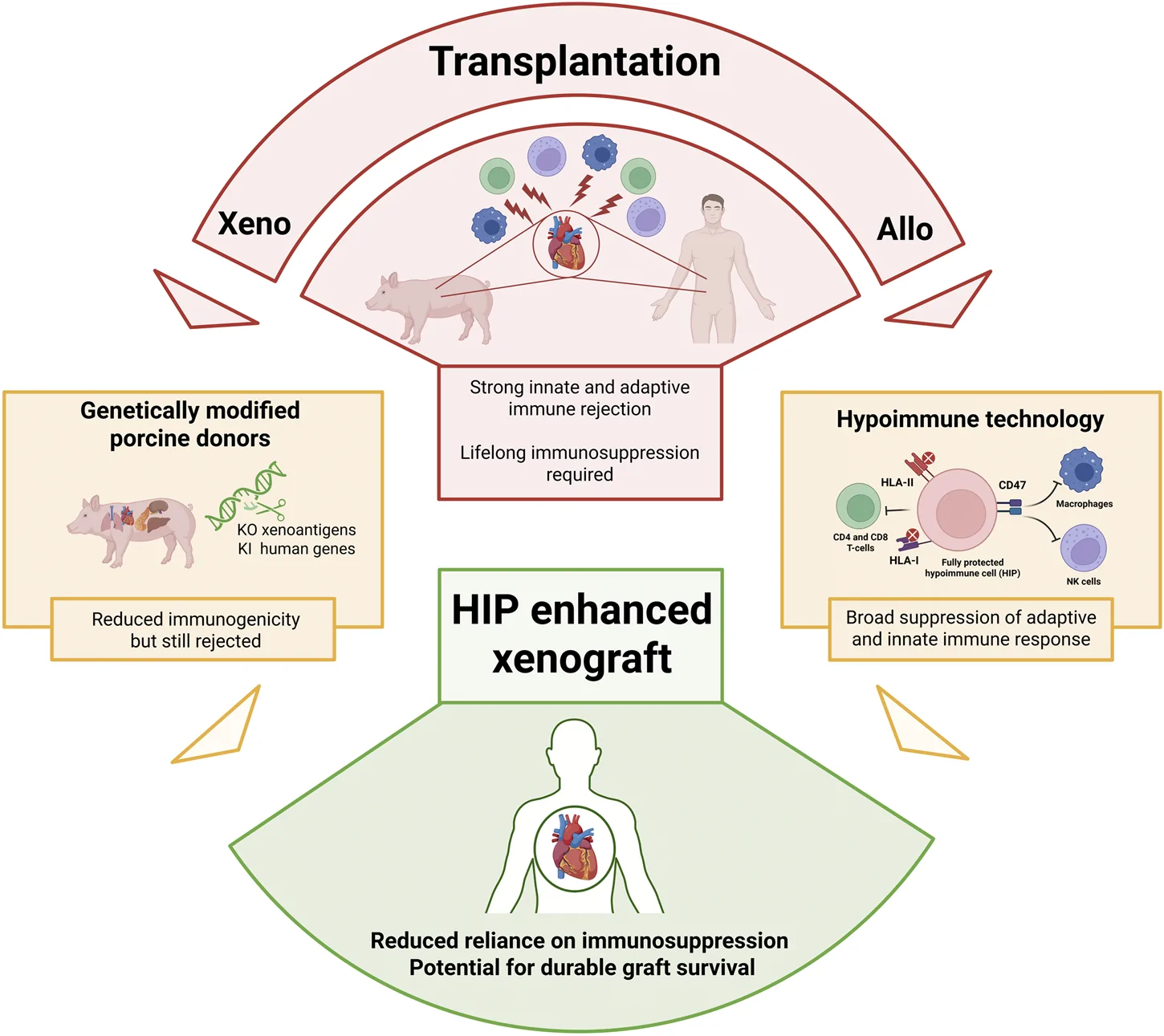

## Introduction

The success of cell, tissue, and organ transplantation has long been constrained by the host immune response, which recognizes donor tissues as non-self and triggers graft rejection (e.g., the pathways of allorecognition and T cell activation) [1]. Lifelong immunosuppressive therapy remains the cornerstone of clinical transplantation, but carries substantial risks, including infection, malignancy, metabolic complications, and chronic allograft dysfunction [2]. At the same time, the increasing demand for transplantable tissues, driven by organ shortages and the rising incidence of chronic diseases including end-stage organ failure [3, 4], has prompted the search for alternative, renewable sources of functional cells and tissues, including those derived from pluripotent stem cells (PSCs) and xenogeneic donors [5, 6]. Regardless of their origin, overcoming immune incompatibility remains a central challenge to achieving durable graft survival without systemic immunosuppression [6].

Recent advances in genome editing have substantially expanded the immune-engineering toolkit, enabling a new class of immune-evasive cell products [7, 8]. By precisely modifying key genes involved in antigen presentation and immune recognition, it is now possible to engineer cells with markedly reduced immunogenicity; whether full immune evasion can be achieved while preserving physiological function remains under investigation [8]. Genome editing technologies such as Clustered Regularly Interspaced Short Palindromic Repeats (CRISPR)–Cas approaches enable precise genetic modifications, including the inactivation of classical human leukocyte antigen (HLA) molecules, modulation of co-stimulatory and inhibitory pathways, and incorporation of immune checkpoint ligands to dampen both innate and adaptive responses [5, 8]. Complementary strategies extend to xenotransplantation, where extensive genome engineering has produced porcine donors lacking xenoantigens and expressing human immunoregulatory molecules [9].

Together, these advances define a rapidly evolving field at the intersection of regenerative medicine [10], transplantation biology, and synthetic immunology. Genome-edited hypoimmune platforms represent an emerging strategy toward ‘off-the-shelf’ cell therapies and broadly compatible tissue grafts, with the potential to transform the clinical management of immune-mediated diseases [6]. Complementary strategies, including localized immune modulation and biomaterial-based immune shielding, are also under active investigation and may contribute to future combinatorial approaches. However, significant challenges remain, including incomplete immune evasion, potential risks of malignant transformation, and the need for precise control of immunomodulatory gene networks [5].

To date, the clinical performance of allogeneic cell-based therapies has lagged behind that of autologous counterparts, largely due to immune rejection and limited persistence of donor cells *in vivo* [11]. This limitation is particularly evident in cancer immunotherapy, where the inability of allogeneic products to achieve long-term engraftment and sustained activity has constrained their therapeutic potential [11]. In contrast, in autoimmune diseases or acute inflammatory conditions, where transient activity rather than long-term persistence may be sufficient, early trials using allogeneic CAR-T cells have demonstrated encouraging results [12].

The next frontier for allogeneic therapies will depend on achieving durable immune evasion and functional stability comparable to autologous cells. Advances in genome editing and synthetic biology now enable the design of allogeneic, immune-evasive cell products with enhanced metabolic fitness, resistance to exhaustion, and improved trafficking within hostile or immunosuppressive environments [6]. However, such extensive engineering is logistically and economically feasible only for standardized, large-scale manufacturing of universal allogeneic products, rather than individualized autologous therapies [6].

In this context, pluripotent stem cell (PSC)-derived immune and parenchymal cells provide a highly scalable platform for iterative, multi-step genome editing and clonal selection [13]. If comprehensive immune cloaking can be reliably achieved, PSC-derived hypoimmune products may ultimately match, or even surpass, autologous therapies, combining broad compatibility with enhanced functional attributes, provided that key safety concerns, including genomic stability and long-term immune surveillance, are adequately addressed [14]. Accordingly, achieving robust and durable immune evasion represents a critical enabling step toward the next-generation of “off-the-shelf” immune and regenerative cell therapies.

Here, we review current genome editing strategies for generating hypoimmune cells and organs, including xenografts. We discuss the molecular targets and design principles underlying immune cloaking, highlight key preclinical and translational advances, and explore the remaining biological and regulatory barriers to clinical implementation. Finally, we suggest future directions for integrating immune engineering with tissue biofabrication and vascularized organ design, toward the development of universally compatible, immune-protected grafts.

## Engineering immune-evasive tissue-based products

The generation of immune-evasive tissue products, hereafter referred to as hypoimmune derivatives, is designed to elude immune recognition by reducing T-cell mediated detection and providing inhibitory signals to the innate immune system. Ideally, transplantation of hypoimmune tissues should avoid activating an immune response and prevent the induction of targeted immunological memory. The recipient’s immune system should not recognize these allogeneic products as non-self; in preclinical models, this has been associated with tolerance of repeated administrations, although long-term clinical data remains limited.

To achieve this immunological bypass, the main challenge is to avoid activation of the adaptive T-cell response. A pivotal factor in this process is the presentation of alloantigens by HLA molecules. To prevent alloantigen presentation through HLA class I, many groups have targeted β2-microglobulin (B2M) [15–17], which is essential for HLA class I molecule expression, or transporter associated with antigen processing 2 (TAP2), a transporter required for peptide loading and presentation via HLA class I. Regarding HLA class II, several cell types in the body constitutively express these molecules; endothelial cells and pancreatic islets can upregulate HLA class II expression during inflammatory conditions [18, 19]. Complete disruption of alloantigen presentation through HLA class I and II depletion prevents T-cell activation and effectively bypasses the adaptive immune response as extensively reviewed in other works [20–24].

**FIGURE 1:**
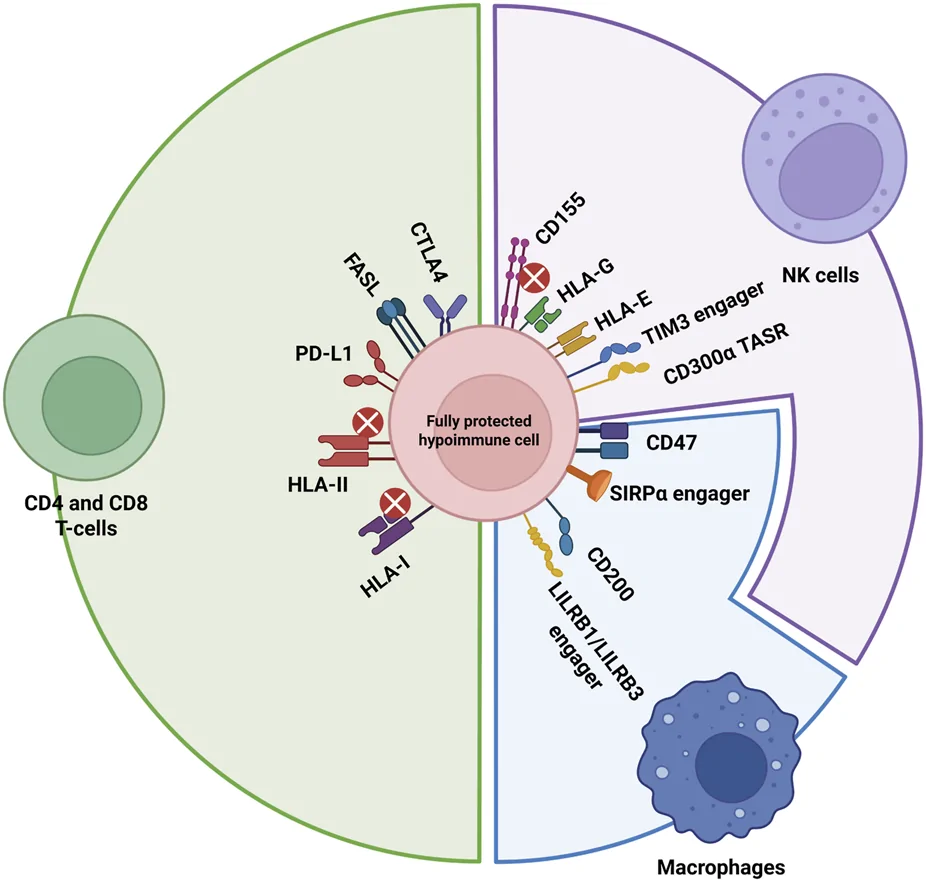
Genome-engineering strategies to generate immune-evasive, cell and tissue-based products. Edits modulating interactions with T cells (green), NK cells (purple), and macrophages (blue) are shown. Red symbols indicate gene knockouts HLA class I, HLA class II, and the activating NK ligand CD155 (PVR), while all other modifications represent knock-in or overexpression of natural or synthetic immunomodulatory ligands to limit immune recognition and clearance. These include PD-L1, CTLA4, and FASL for T cells; HLA-G, HLA-E, and TIM3 and CD300a engagers for NK-specific inhibition; and CD200 and LILRB1/LILRB3 engagers for macrophages, alongside CD47 and SIRPα engagers, which act on both NK cells and macrophages to deliver a shared “do not eat me”/ “do not kill me” inhibitory signal. Together, these edits illustrate the multi-layered strategy required to protect a single hypoimmune cell from adaptive and innate rejection simultaneously. Figure was created in BioRender (https://BioRender.com).

However, adaptive immune cells are not the only mediators of graft rejection. Innate immune components, such as NK cells and macrophages, can independently sense HLA deletion, triggering the “missing self-response” and mediate a cytotoxic response [25]. NK cells express a broad repertoire of activating and inhibitory surface receptors whose balance governs cytotoxic output and immunological engagement (Figure 1). SIRPα has been identified as a potent inhibitory immune checkpoint for NK cells and the overexpression of its ligand, CD47, results in potent inhibition of NK cells [26]. Accordingly, effective immune evasion in engineered cell therapies requires a multi-layered strategy. HLA-depleted cells rely on high CD47 expression, well above normal levels, to escape NK-cell attacks, exploiting the threshold-dependent “on/off” nature of NK cytotoxicity. This effect can be amplified through broadly expressed inhibitory receptors such as SIRPα, TIM3, and CD300a [27, 28], or by overexpressing HLA-E [16] and HLA-G [29] to target specific NK subsets. Removal of activating ligands, such as the poliovirus receptor (PVR/CD155), may further shift the balance toward inhibition, although this alone is insufficient, as shown in iPSC-derived T cells [16].

Partial retention of selected HLA molecules can reduce T-cell activation while preserving innate immune protection [30], yet this approach depends on patient matching, requires cell banks, and introduces logistical challenges. In HLA-depleted cells, combining checkpoint modulation, interference with antigen presentation, and secretion of immune-regulatory factors can further dampen rejection. Antibody-mediated responses can also be mitigated through engineered expression of modified CD64 or IgG-degrading enzymes, thereby broadening protection without compromising resistance to cellular immunity [31]. However, clinical experience demonstrates that even HLA-matched grafts require immunosuppression, emphasizing the complexity of immune recognition and the need for caution when developing universal immune-evasion strategies.

HLA class I and II knockdown is not the only proposed strategy. Harding et al. worked on possible combinatorial overexpression of immunomodulatory factors such as PD-L1, CD200, CD47, FASL, SERPINB9, CCL21, MFGE8 and HLA-G without HLA class I and II knockout in hESCs to generate cloaked cells. Upon differentiation in retinal pigmented epithelial cells and coculture with allogeneic human PBMCs, cloaked cells were able to persist and to not elicit an allogeneic response. In addition, the same strategy was used to generate cloaked tissues in a subcutaneous site that serves as an immune-privileged site that protects allogeneic and xenogeneic uncloaked cells from rejection in immunocompetent hosts demonstrating the long-term survival of a solid tissue allograft without the need for systemic immunosuppression [32].

## Regulatory considerations for clinical translation

Regulatory frameworks governing the clinical translation of genetically engineered hypoimmune pluripotent stem cell (HIP)-derived products and xenogeneic grafts are rapidly evolving across multiple jurisdictions to ensure safety, efficacy, product consistency, and long-term patient monitoring.

In the European Union (EU), genetically modified HIP products are classified as Advanced Therapy Medicinal Products (ATMPs) under Regulation (EC) No 1394/2007 and are regulated by the European Medicines Agency (EMA), which provides guidance on genomic stability, potency testing, biodistribution, tumorigenicity assessment, pharmacovigilance, and long-term follow-up requirements for advanced cellular therapies [33]. In addition, products involving genetically modified organisms (GMOs) must comply with both EU-wide and national biosafety regulations under the EU GMO framework, adding further complexity to clinical development.

In the United States, cell and gene therapies are regulated by the Food and Drug Administration (FDA), primarily through the Center for Biologics Evaluation and Research (CBER). Human cells, tissues, and cellular and tissue-based products are regulated under 21 CFR Parts 1270 and 1271, while more substantially manipulated or genetically modified products require Investigational New Drug (IND) applications and Biologics License Applications (BLAs) before clinical use. FDA guidance emphasizes donor eligibility, genomic integrity, off-target editing analysis, sterility, manufacturing reproducibility, potency assays, and prolonged post-transplant surveillance, particularly for genome-edited and stem cell-derived products [34]. Furthermore, the FDA has established a regenerative medicine framework to support the development of advanced cellular therapies while maintaining rigorous standards for safety and efficacy [35, 36].

Similarly, in the United Kingdom, Advanced Therapy Medicinal Products are regulated by the Medicines and Healthcare products Regulatory Agency (MHRA), which continues to maintain a framework closely aligned with EU ATMP principles while developing independent innovation pathways and specialized guidance for GMP-compliant manufacturing, vector characterization, assay validation, and release testing [36, 37].

Outside Europe and the United States, regulatory frameworks across Canada, Australia, and Asia continue to evolve in response to advances in regenerative medicine, genome engineering, and xenotransplantation, reflecting a global effort to balance innovation with rigorous safety and quality standards. In Canada, cell and gene therapies are regulated by Health Canada under the Food and Drugs Act, following a risk-based framework broadly aligned with FDA and EMA principles that emphasizes GMP compliance, donor eligibility, potency and identity testing, genomic stability, traceability, and long-term safety monitoring; recent regulatory initiatives have further sought to facilitate the clinical development of innovative therapies without compromising these standards. Australia follows a comparable risk-based approach through the Therapeutic Goods Administration (TGA). TGA classifies genetically modified cellular products as biologicals or gene therapies depending on the degree of manipulation and intended clinical use, and requires GMP-compliant manufacturing, validated potency assays, donor screening, genomic integrity assessment, and comprehensive pharmacovigilance; growing alignment with international standards has facilitated Australia’s participation in global clinical development programs. Among Asian countries, Japan offers a notably different model: through the Pharmaceuticals and Medical Devices Agency (PMDA) and the Act on the Safety of Regenerative Medicine, it has introduced conditional and time-limited approval pathways that allow earlier clinical access following demonstration of preliminary safety and probable efficacy [36]. This framework has already accelerated the translation of induced pluripotent stem cell (iPSC)-derived therapies and may offer a regulatory template for future hypoimmune cellular products [36]. Taken together, while Canada and Australia largely mirror the risk-based, evidence-heavy approach of the FDA and EMA, Japan’s conditional-approval pathway illustrates an alternative route to earlier patient access, a distinction directly relevant to how hypoimmune platforms might reach the clinic outside Europe and the US.

Alongside national regulatory agencies, international scientific organizations are actively playing a major role in defining governance frameworks for xenotransplantation. The International Xenotransplantation Association (IXA) has published multiple consensus statements addressing ethical considerations, clinical trial design, microbiological safety, source animal standards, infectious disease surveillance, informed consent, traceability, and long-term recipient monitoring [38, 39]. In addition, the Changsha Communiqué [40] emphasized the urgent need for harmonized international oversight, coordinated bio-surveillance programs, standardized pathogen screening, transparent clinical reporting, and global data sharing to address zoonotic and biosafety risks associated with xenogeneic products [40]. These recommendations are particularly relevant for HIP-engineered xenografts and stem cell-derived tissues, where extensive genome editing may alter immune recognition while simultaneously complicating pathogen detection and long-term immunological surveillance.

Nevertheless, substantial regulatory challenges remain due to differences in regulatory terminology, approval pathways, GMO oversight, and long-term follow-up requirements. Limited international harmonization creates significant burdens for developers pursuing global clinical translation of hypoimmune cell products.

In parallel with regulatory approval pathways, successful clinical translation will critically depend on the establishment of robust manufacturing and quality control strategies. Clinical-grade production of hypoimmune cellular products requires GMP-compliant workflows for genome editing, clonal selection, expansion, differentiation, and cryopreservation while maintaining genomic stability and phenotypic consistency throughout manufacturing. Rigorous release criteria will likely require verification of intended genetic modifications, assessment of off-target events, confirmation of stable expression of immunomodulatory transgenes, sterility testing, genomic integrity analysis, tumorigenicity assessment, and validated potency assays demonstrating durable immune evasion. Furthermore, scalable production platforms based on well-characterized pluripotent stem cell master and working cell banks must ensure reproducibility, traceability, and batch-to-batch consistency. Collectively, these considerations illustrate that successful clinical implementation of hypoimmune cellular therapies will require not only advances in genome engineering, but also internationally harmonized regulatory policies, standardized manufacturing platforms, and coordinated long-term biosafety surveillance.

## Hypoimmune platform technology: design principles and preclinical evidence

The hypoimmune platform (HIP) is one suggested approach to render engineered cells resistant to both innate and adaptive immune responses [7]. The platform combines knockout of genes encoding β2-microglobulin (B2M) and class II transactivator (CIITA), to eliminate expression of HLA class I and II molecules, together with overexpression of CD47 which delivers a potent “do not eat me” signal that inhibits macrophage- and NK-cell–mediated cytotoxicity [26]. Although CD47’s role in preventing phagocytosis has been long established [41], its efficacy in suppressing NK-cell activity via the SIRPα pathway has only recently been clarified [26], with preclinical data suggesting broad inhibition across major NK subsets, though systematic evaluation across all human NK cell compartments is ongoing. In preclinical models, HIP engineering of primary human CAR T cells or iPSCs generates heterogeneous populations, yet only fully HIP-edited cells survive in environments where HLA-depleted or partially edited cells are eliminated, demonstrating their ability to withstand active immune rejection. Clinical experience with HIP CD19 CAR-T cells mirrors these findings: fully edited cells avoid T- and NK-cell mediated killing and do not induce antibody responses, whereas unedited or partially edited populations are rapidly rejected. Notably, current evidence suggests that HIP cells may not readily induce immunological memory. If confirmed, this feature could enable repeated dosing strategies, an important advantage over autologous therapies, which could be limited to single administration due to the induction of immunological memory reducing overall survival of the transplanted cells [30]. By overcoming both innate and adaptive immune barriers, HIP technology offers a versatile platform for allogeneic cell therapies. Its durability has been demonstrated in preclinical models of beta cell replacement for T1D. HIP-engineered iPSC-derived and primary human islets survive, engraft, and restore glycemic control in fully allogeneic, immunocompetent humanized mice without triggering immune responses [8]. Importantly, HIP cells also resist autoimmune attack, as demonstrated in T1D-derived autologous humanized models [7]. Advances in gene-editing techniques now enable HIP modification of primary human islets, which retain superior endocrine function compared to iPSC-derived counterparts while maintaining immune evasion [7]. Nonhuman primate studies confirmed long-term engraftment and function of HIP-edited allogeneic islets for over 6 months without immunosuppressive therapy [42]. While HIP offers a powerful strategy to evade both innate and adaptive immune rejection, the long-term safety of HLA-depleted cells remains incompletely understood. Preclinical models in MHC class I–deficient mice show impaired viral clearance, though these animals also have severe CD8 T-cell deficiencies, limiting direct translation to immunocompetent humans [43, 44]. In real-world applications, the number of transplanted hypoimmune cells is low, and recipients maintain fully functional immune systems, yet the possibility that HLA-deficient cells may impair intracellular antigen presentation and thus serve as undetected reservoirs for persistent viruses or intracellular pathogens cannot be excluded and warrants prospective clinical monitoring. The recently developed HIP mouse, globally deficient in MHC class I and II and overexpressing CD47, demonstrated normal viability, fertility, and no increased susceptibility to infections or tumors, suggesting limited intrinsic risk [45]. Nevertheless, potential concerns remain, including the unknown effects of long-term persistence, unexpected interactions with host immunity, and the theoretical risk of harboring pathogens undetected [45]. Careful pre-clinical evaluation and clinical monitoring will be essential to ensure safety as these platforms advance toward therapeutic use.

## HIP islet cell transplantation: a clinical proof-of-concept

Allogeneic and xenogeneic beta cell replacement [46, 47] offers a promising approach to restore glycemic control in patients with diabetes, potentially overcoming the limitations of conventional insulin therapy [48] and autologous cell transplantation. Early clinical experiences with PSC-derived islets, including both autologous and allogeneic approaches, have demonstrated the capacity to improve C-peptide levels, improve glycemic control, and achieve insulin independence in some cases [47, 49, 50]. However, in these cases, recipients required systemic immunosuppression due to allogeneic nature of the graft or prior organ transplants, masking the true immune response and highlighting the substantial challenge of achieving durable engraftment in immunocompetent patients [49]. Partial immune-evasion strategies, including selective HLA class I or I/II depletion, PD-L1 overexpression, or localized secretion of immunomodulatory factors such as IL-2 mutein, TGF-β or IL-10 have shown inconsistent results in preclinical models, often failing to fully protect transplanted cells from NK-mediated or autoimmune attack [18]. These limitations have motivated the development of more comprehensive immune-engineering approaches such as the HIP islet cells, which simultaneously target multiple immune recognition pathways as mentioned before [7, 8, 42]. The translational potential of HIP platforms has been explored in an initial human case report. HIP-engineered allogeneic islets were transplanted into a patient with type 1 diabetes without immunosuppression. Four weeks post-transplant, functional insulin secretion was evident through circulating C-peptide and mixed meal stimulated responses, and MRI confirmed graft survival. No immune activation or safety signals were detected over the reported follow-up period, providing preliminary evidence that comprehensive immune evasion may be achievable in selected clinical settings; however, these observations derive from a single recipient with only short-term (4-week) follow-up and limited immunological characterization, longer follow-up and larger cohorts are needed to assess durability and generalizability of the platform [51].

These findings suggest the potential of allogeneic endocrine cell therapy. HIP platforms may overcome key limitations of partial immune protection strategies, and preliminary data indicate a potential pathway toward immunosuppression-free treatment for diabetes; this remains to be validated in adequately powered clinical trials. By enabling repeated dosing, sustained glycemic control, and protection from both allorejection and autoimmunity, allogeneic HIP-engineered cells represent a promising advance in regenerative medicine, with early data supporting their therapeutic potential in metabolic disease.

## HIP technology in xenotransplantation

Advances in genome editing have substantially accelerated xenotransplantation research, enabling the development of multi-edited porcine donors with reduced immunogenicity [52]. With up to ten targeted genetic modifications, including knockouts of major carbohydrate antigens, the introduction of human complement regulatory transgenes, and other immune-evasive edits, porcine organs have become increasingly compatible with the human immune system. These typically include knockout of the major carbohydrate xenoantigens (GGTA1, CMAH, B4GALNT2/2L) and of the growth hormone receptor (GHR), together with knock-in of human complement- and coagulation-regulatory transgenes (CD46, CD55, THBD, EPCR), the anti-inflammatory and anti-thrombotic factor TNFAIP3, the cytoprotective enzyme HMOX1, and CD47 to limit macrophage-mediated clearance [9]. Preclinical studies and early clinical applications [53] involving hearts [54], kidneys [55], and livers [56] have demonstrated feasibility, yet even highly engineered organs remain vulnerable to residual immune-mediated injury, including antibody-mediated rejection, micro-vascular inflammation, NK cell–mediated cytotoxicity, and macrophage-driven clearance [9, 57]. These observations underscore that, despite substantial advances, additional strategies are needed to achieve long-term graft survival.

**FIGURE 2:**
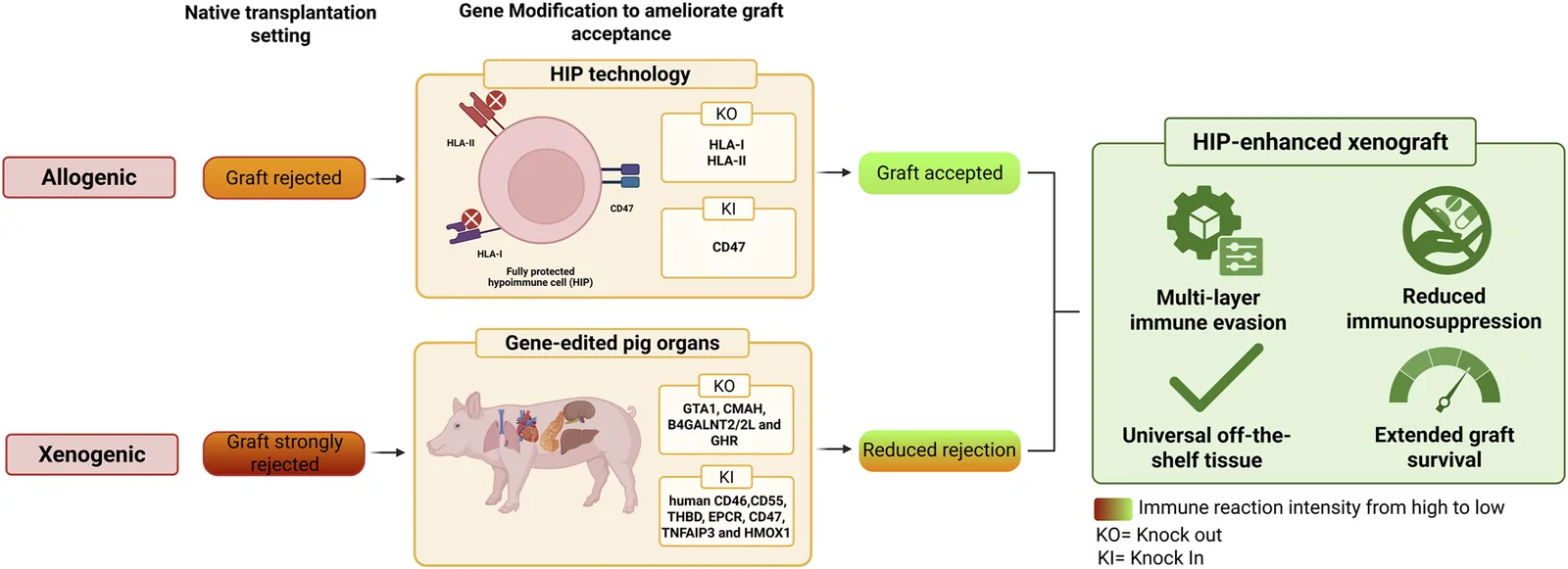
HIP-enhanced xenograft. Conventional allogeneic grafts are rejected, and xenogeneic grafts are subject to rapid immune destruction driven by combined adaptive and innate responses. Hypoimmune platform (HIP) engineering combines HLA class I and II knockout with CD47 overexpression, thereby blocking T-cell recognition and suppressing NK cell- and macrophage-mediated clearance. In parallel, gene-edited porcine organs incorporating knockout of the major xenoantigens and the growth hormone receptor (GGTA1, CMAH, B4GALNT2/2L, GHR) together with human complement- and coagulation-regulatory and cytoprotective transgenes (CD46, CD55, THBD, EPCR, CD47, TNFAIP3, HMOX1) exhibit reduced immunogenicity. The integration of porcine genome editing with HIP immune cloaking may enable the generation of universally compatible xenografts capable of long-term survival with reduced and potentially, in selected contexts, no systemic immunosuppression. Figure was created in BioRender (https://BioRender.com).

In this context, HIP engineering offers a powerful complementary approach. HIP platforms combine targeted depletion of MHC molecules with high CD47 expression, providing broad protection from both adaptive and innate immune attacks [6]. By inhibiting NK cell cytotoxicity, preventing macrophage phagocytosis, targeting polymorphonuclear cells [57] and reducing T and B cell activation, HIP modifications add an additional layer of immune protection that could be particularly valuable in xenotransplantation, where multiple immune pathways act in parallel to reject grafts [52]. Preclinical studies suggest that HIP-modified cells and tissues can survive in highly immunogenic environments, resisting both early innate responses and subsequent adaptive immune recognition [7, 8, 42, 57]. HIP engineering may be integrated with existing porcine genetic modifications to further reduce antigenicity, mitigate complement and antibody-mediated injury, and enhance graft longevity [9] (Figure 2). In this direction, triple (GGTA1, CMAH, B2M) modified pigs expressing Swine Leucocyte Antigen class I at low level were generated. The KO of SLA class I showed to prevent human CD8 T cell activation in xenotransplantation setting demonstrating the feasibility of the approach [58]. However, complete SLA class I deficiency in porcine donors was associated with impaired immune development, including reduced CD4^+^ and CD8^+^ T-cell compartments, hypogammaglobulinemia, and decreased survival. Thus, specific housing and animal care conditions should be considered for these animals [58].

Despite these advances, several critical challenges remain.

The risk of viral transmission, particularly from porcine endogenous retroviruses (PERVs) and porcine cytomegalovirus (PCMV), represents a key safety concern. Inactivation of PERV sequence, normally integrated into the porcine genome, via CRISPR–Cas technology, has been demonstrated to be highly successful generating inactivated donor lines [59, 60]. In contrast PCMV still represents a concern since transmission to humans after transplantation of pig heart was observed and contributed to the death of the recipient [61, 62]. Thus, other mitigation strategies like stringent screening, and long-term recipient monitoring are needed as well as the use of donor animals raised in GMP facilities under strict, pathogen-free, and traceable conditions and thus suitable for human use. This strategy may complement donor genome-editing approaches or prophylactic anti-infective therapies [63]. HLA-deficient HIP grafts may complicate detection of infected cells due to impaired antigen presentation, adding additional safety challenges [64]. Adaptive changes in innate immunity or recognition of residual xenoantigens could emerge over time, necessitating long-term monitoring. Moreover, manufacturing, quality control, and regulatory challenges increase as HIP engineering is combined with multi-gene xenotransplantation platforms. Despite these hurdles, HIP approaches offer the potential to generate xenogeneic grafts with durable, multi-layered immune evasion, potentially reducing reliance on systemic immunosuppression, pending validation in well-controlled clinical trials.

The potential of combining genome-edited porcine organs with HIP modifications is considerable, though it currently rests primarily on early preclinical evidence and remains largely unproven in clinical settings. Such an approach could extend graft survival, allow for repeated graft administration, and broaden the applicability of xenotransplantation to patients with metabolic, endocrine, or organ failure. By addressing both innate and adaptive immune barriers simultaneously, HIP-enhanced xenografts may bridge the remaining gap between experimental success and clinically viable organ replacement therapies, potentially offering a pathway toward safer, more durable therapies. In the long term, these advances may contribute to addressing the global shortage of transplantable organs, provided that remaining immunological, safety, and regulatory challenges are successfully resolved.
